# Effect of guided tissue regeneration using bioresorbable membranes on periodontal regeneration in grade II furcation defects

**DOI:** 10.6026/973206300223186

**Published:** 2026-05-31

**Authors:** Rajesh Lohiya, Kiran Dodani, Ankur singh Rajpoot, Pallavi Goswami, Anup Shelke, Shilpa Jaiswal

**Affiliations:** 1 Department of Periodontology and Implant Dentistry, RKDF Dental College and Research Centre, Bhopal, Madhya Pradesh, India; 2 Department of Periodontology, RKDF Dental College and Research Centre, Bhopal, Madhya Pradesh, India; 3 Department of Periodontology, Dr HSRSM Dental College and Hospital, Hingoli, Maharashtra, India

**Keywords:** Guided tissue regeneration (GTR), bioresorbable membrane, furcation defects, periodontal regeneration, collagen membrane

## Abstract

Grade II furcation defects pose a significant therapeutic challenge in periodontology due to their complex anatomy and limited accessibility for effective regeneration. Therefore, it is of interest to evaluate the efficacy of guided tissue regeneration (GTR) using a bioresorbable collagen membrane compared to open flap debridement (OFD) alone in 48 patients with mandibular molar Grade II furcation defects. Clinical parameters (probing depth, clinical attachment level, horizontal probing depth and gingival recession) and radiographic bone fill were assessed at baseline and 6 months postoperatively. The GTR group demonstrated significantly greater improvements in PD reduction (3.42 ± 0.78 mm vs 2.18 ± 0.65 mm), CAL gain (2.89 ± 0.72 mm vs 1.54 ± 0.58 mm), HPD reduction (2.67 ± 0.54 mm vs 1.23 ± 0.48 mm) and bone fill (48.6 ± 12.3% vs 22.4 ± 9.8%) compared to OFD (p<0.001). GTR with a bioresorbable collagen membrane provides superior clinical and radiographic outcomes, supporting its use as an effective regenerative approach for Grade II furcation defects.

## Background:

Periodontitis is a chronic inflammatory disease affecting the supporting structures of teeth, representing a significant global health burden with prevalence rates exceeding 45% in adult populations worldwide [1]. Among the various manifestations of periodontal destruction, furcation involvement presents particularly challenging clinical scenarios due to the complex anatomical configuration of the interradicular region [2]. Furcation defects are classified according to the degree of horizontal attachment loss, with Grade II defects characterized by horizontal bone loss exceeding one-third of the tooth width but not encompassing the total width of the furcation area [3]. The management of furcation defects has historically been associated with unpredictable outcomes, primarily due to limited accessibility for instrumentation and the intricate root morphology of multi-rooted teeth [4]. Conventional therapeutic approaches, including scaling and root planing or open flap debridement, often result in repair rather than true regeneration, characterized by long junctional epithelium formation rather than new attachment with cementum, periodontal ligament and alveolar bone [5]. Guided tissue regeneration emerged as a revolutionary concept in periodontal therapy demonstrated that selective cell repopulation, could achieve true periodontal regeneration [6]. The GTR principle is based on excluding epithelial and gingival connective tissue cells from the healing wound while permitting periodontal ligament and bone cells to repopulate the defect space [7]. This is accomplished through the placement of barrier membranes that create a protected space for regeneration.

Initial GTR procedures utilized non-resorbable expanded polytetrafluoroethylene (ePTFE) membranes, which demonstrated favorable regenerative outcomes but required a second surgical procedure for membrane removal [8]. Subsequently, bioresorbable membranes were developed to eliminate the need for secondary surgery, thereby reducing patient morbidity, surgical costs and potential complications associated with membrane retrieval [9]. Among bioresorbable materials, collagen membranes have gained widespread acceptance due to their excellent biocompatibility, hemostatic properties and ability to promote wound healing [10]. Several clinical studies have investigated the efficacy of GTR in furcation defects, with varying results attributed to differences in membrane types, defect configurations and surgical techniques [11, 12]. However, considerable heterogeneity exists among studies regarding membrane materials and outcome measures. Despite substantial evidence supporting GTR, gaps remain in understanding the optimal application of bioresorbable membranes specifically in mandibular Grade II furcation defects. Furthermore, there is a need for standardized clinical protocols and comprehensive outcome assessments incorporating both clinical and radiographic parameters [13]. Therefore, it is of interest to evaluate and compare the clinical and radiographic outcomes of GTR using bioresorbable collagen membranes versus open flap debridement alone in the treatment of Grade II mandibular molar furcation defects over a 6-month follow-up period.

## Materials and Methods:

## Study design and ethical considerations:

This randomized controlled clinical trial was conducted at the Department of Periodontology between August 2024 and August 2025.

## Sample size calculation:

Sample size was calculated based on previous studies reporting mean clinical attachment level gains following GTR procedures. Assuming a mean difference of 1.2 mm between groups, standard deviation of 1.0 mm, alpha error of 0.05 and power of 80%, a minimum of 20 patients per group was required. To account for potential dropouts, 24 patients were enrolled in each group.

## Patient selection:

## Inclusion criteria:

[1] Age between 30-60 years

[2] Diagnosis of chronic periodontitis with Grade II mandibular molar furcation involvement

[3] Probing depth ≥5 mm at furcation site

[4] Horizontal probing depth of 3-6 mm

[5] Good systemic health

[6] Commitment to follow-up visits

## Exclusion criteria:

[1] Systemic diseases affecting periodontal healing (diabetes mellitus, immunocompromising conditions)

[2] Smoking habit (current or former within 5 years)

[3] Pregnancy or lactation

[4] Previous periodontal surgery at the selected site

[5] Teeth with Grade III mobility

[6] Use of antibiotics or anti-inflammatory drugs within 3 months

## Randomization and blinding:

Eligible patients were randomly assigned to either the test group (GTR + OFD) or control group (OFD alone) using computer-generated random numbers in sealed opaque envelopes. The examiner performing clinical measurements was blinded to treatment allocation.

## Pre-surgical phase:

All patients underwent comprehensive oral hygiene instructions and full-mouth scaling and root planing using ultrasonic scalers and hand instruments. Patients were re-evaluated after 4-6 weeks and only those demonstrating adequate plaque control (plaque index <20%) proceeded to surgical intervention.

## Clinical parameters:

Clinical measurements were recorded at baseline (immediately before surgery) and at 6 months post-surgery using a UNC-15 periodontal probe by a calibrated examiner (intra-examiner reliability: ICC=0.92). Parameters included:

[1] Probing depth (PD)

[2] Clinical attachment level (CAL)

[3] Horizontal probing depth (HPD) using a Nabers probe

[4] Gingival recession (GR)

[5] Plaque index (PI)

[6] Gingival index (GI)

## Radiographic assessment:

Standardized periapical radiographs were obtained at baseline and 6 months using the paralleling technique with customized film holders. Radiographic bone fill was calculated as the percentage change in radiolucency within the furcation area using digital image analysis software.

## Surgical procedure:

All surgeries were performed by a single experienced periodontists under local anesthesia (2% lignocaine with 1:80,000 adrenalines). Following intrasulcular incisions, full-thickness mucoperiosteal flaps were elevated to expose the furcation defect. Thorough debridement of the defect was performed, including removal of granulation tissue and root planing using Gracey curettes.

## Test group:

Following debridement, a bioresorbable collagen membrane (cross-linked porcine collagen, 20x30 mm) was trimmed and adapted to completely cover the furcation defect, extending 2-3 mm beyond the defect margins. The membrane was secured using resorbable sutures.

## Control group:

[1] Flaps were repositioned and sutured following debridement without membrane placement.

[2] Both groups received primary closure using 4-0 silk sutures with interrupted suturing technique.

## Post-surgical protocol:

Patients were prescribed amoxicillin 500 mg three times daily for 5 days and ibuprofen 400 mg as needed. Chlorhexidine gluconate 0.12% mouthrinse was prescribed twice daily for 4 weeks. Sutures were removed at 10-14 days. Patients were recalled at 1, 3 and 6 months for professional maintenance.

## Statistical analysis:

Data were analyzed using SPSS version 26.0. Normality was assessed using Shapiro-Wilk test. Intragroup comparisons were performed using paired t-tests, while intergroup comparisons utilized independent sample t-tests. Chi-square test was used for categorical variables. Statistical significance was set at p<0.05.

## Results:

Forty-eight patients completed the 6-month follow-up period, with 24 patients each in the test and control groups. The test group included 13 males and 11 females, while the control group also included 13 males and 11 females. The mean age was 45.2 ± 7.9 years in the test group and 44.1 ± 8.6 years in the control group, with no statistically significant difference between groups (p = 0.648). Baseline clinical parameters, including Plaque Index, Gingival Index, probing depth, clinical attachment level, horizontal probing depth, and gingival recession, were comparable between the two groups, indicating adequate baseline homogeneity (Table 1). Both groups showed improvement in periodontal clinical parameters after 6 months. The mean probing depth reduced from 6.54 ± 0.86 mm to 3.12 ± 0.58 mm in the test group, whereas it reduced from 6.38 ± 0.92 mm to 4.21 ± 0.72 mm in the control group. The mean probing depth reduction was significantly greater in the test group (3.42 ± 0.78 mm) compared to the control group (2.18 ± 0.65 mm) and the intergroup difference was statistically significant (p < 0.001). Clinical attachment level also improved in both groups. In the test group, mean CAL reduced from 7.21 ± 0.94 mm at baseline to 4.32 ± 0.76 mm at 6 months, with a mean gain of 2.89 ± 0.72 mm. In the control group, CAL reduced from 7.08 ± 0.88 mm to 5.54 ± 0.82 mm, with a mean gain of 1.54 ± 0.58 mm. The difference in CAL gain between the two groups was statistically significant, favoring the test group (p < 0.001). Horizontal probing depth showed a marked reduction in the test group. The mean HPD decreased from 4.42 ± 0.68 mm to 1.75 ± 0.52 mm in the test group, while in the control group it decreased from 4.29 ± 0.72 mm to 3.06 ± 0.64 mm. The mean HPD reduction was 2.67 ± 0.54 mm in the test group and 1.23 ± 0.48 mm in the control group, showing a statistically significant intergroup difference (p < 0.001). Gingival recession increased slightly in both groups during the follow-up period. The mean change in gingival recession was 0.54 ± 0.38 mm in the test group and 0.67 ± 0.44 mm in the control group. However, this difference was not statistically significant (p = 0.274). At 6 months, gingival recession values were also comparable between the test group and control group (1.21 ± 0.42 mm vs. 1.38 ± 0.52 mm; p = 0.218) (Table 2). Radiographic evaluation demonstrated superior bone regeneration in the test group. Baseline bone loss was comparable between the test and control groups (52.4 ± 14.6% vs. 50.8 ± 13.2%; p = 0.684). At 6 months, bone loss reduced to 26.8 ± 11.4% in the test group and 39.4 ± 12.8% in the control group, showing a statistically significant difference (p < 0.001). Radiographic bone fill was significantly greater in the test group (48.6 ± 12.3%) compared to the control group (22.4 ± 9.8%) (p < 0.001). With respect to furcation treatment success, complete closure or conversion to Grade 0 was observed in 7 patients (29.2%) in the test group compared to 1 patient (4.2%) in the control group and this difference was statistically significant (p = 0.018). Partial improvement to Grade I was observed in 14 patients (58.3%) in the test group and 10 patients (41.7%) in the control group; however, this difference was not statistically significant (p = 0.247). No improvement was recorded in only 3 patients (12.5%) in the test group compared to 13 patients (54.2%) in the control group, showing a statistically significant difference (p = 0.002) (Table 3). Membrane exposure occurred in 2 patients in the test group during the early healing period, corresponding to 8.3% of the test group, not 58.3%. These cases were managed conservatively using chlorhexidine application and close postoperative monitoring. No infections or major adverse effects were observed in either group during the study period.

## Discussion:

The present randomized controlled clinical trial demonstrated that guided tissue regeneration using bioresorbable collagen membranes produced significantly better clinical and radiographic outcomes than open flap debridement alone in the management of Grade II mandibular molar furcation defects. The findings support the biological principle of guided tissue regeneration, in which a barrier membrane prevents rapid epithelial downgrowth and allows selective repopulation of the defect area by periodontal ligament cells, cementoblast and bone- forming cells, thereby promoting periodontal regeneration rather than simple repair. The greater probing depth reduction observed in the test group indicates improved resolution of periodontal pocketing after regenerative therapy. In the present study, the mean probing depth reduction was 3.42 mm in the GTR group compared with 2.18 mm in the control group. This difference suggests that the placement of a bioresorbable collagen membrane provided additional regenerative benefit beyond conventional open flap debridement. Similar clinical improvements have been reported in previous studies evaluating barrier membrane-assisted regenerative therapy for furcation defects [14]. The greater reduction in probing depth may be attributed to improved defect isolation, stabilization of the wound and formation of new periodontal attachment within the furcation area. Clinical attachment level gain was also significantly higher in the test group. The GTR group showed a mean CAL gain of 2.89 mm, whereas the control group showed a gain of 1.54 mm. This finding is clinically important because CAL gain reflects improvement in periodontal support and functional attachment. The superior CAL gain in the test group supports the regenerative potential of collagen membrane-assisted therapy. The use of bioresorbable membranes offers an additional advantage because it avoids the need for a second surgical procedure for membrane removal, thereby reducing patient morbidity and improving clinical acceptability [14]. Horizontal probing depth reduction was one of the most important findings in the present study. The test group showed a mean HPD reduction of 2.67 mm compared with 1.23 mm in the control group. Since Grade II furcation defects primarily involve horizontal loss of interradicular bone, improvement in horizontal probing depth represents a meaningful clinical outcome. The radiographic bone fill of 48.6% observed in the test group further supports the effectiveness of collagen membrane-assisted GTR in promoting bone regeneration within furcation defects. Previous evidence has reported comparable horizontal bone gain using bioresorbable membrane materials, supporting the clinical relevance of the present findings [15]. The improvement in furcation status further confirms the regenerative benefit of GTR. Complete closure of the furcation defect was observed in 29.2% of sites in the test group compared with only 4.2% in the control group. In addition, partial improvement to Grade I was observed in 58.3% of test sites. Overall, conversion of Grade II defects to Grade I or Grade 0 was achieved in 87.5% of GTR-treated sites. This high rate of clinical improvement indicates that bioresorbable collagen membranes may provide predictable outcomes in carefully selected mandibular Grade II furcation defects. These findings are consistent with earlier evidence showing favorable success rates for GTR in Grade II furcation lesions [16]. The superior performance of collagen membranes may be explained by their biological and handling properties. Collagen membranes are biocompatible, hemostatic, easy to adapt and capable of supporting wound stabilization during the early healing phase. They also possess favorable tissue integration properties and may enhance fibroblast migration and clot stabilization. The barrier function provided by the membrane during the initial 4-6 weeks is important because early wound stability is essential for periodontal regeneration. These characteristics make collagen membranes suitable alternatives to non-resorbable membranes in routine clinical use [17]. Membrane exposure was observed in 2 patients, corresponding to 8.3% of the test group. These exposures occurred during the early healing period and were managed conservatively using chlorhexidine application and close postoperative monitoring. No infection or major adverse effect was observed. The limited clinical impact of membrane exposure in the present study may be related to the favorable tissue response associated with collagen membranes. Unlike non-resorbable barriers, exposed collagen membranes may be better tolerated and may not always require immediate surgical intervention [18]. The control group also demonstrated improvement after open flap debridement, although the magnitude of improvement was lower than that of the test group. This suggests that open flap debridement can reduce inflammation, improve access for root surface debridement and support soft tissue healing. However, the healing following OFD alone is generally considered to represent periodontal repair, often through formation of a long junctional epithelium, rather than true regeneration of cementum, periodontal ligament and alveolar bone. The significantly greater clinical and radiographic improvements observed in the GTR group suggest that barrier membrane therapy promoted a more regenerative pattern of healing [19]. Several factors may have contributed to the favorable outcomes observed in the test group. Proper case selection, adequate plaque control, precise flap management, complete defect debridement, stable membrane placement and strict postoperative maintenance are important determinants of regenerative success. In the present study, standardized surgical and postoperative protocols were followed, which likely enhanced wound stability and minimized contamination during the healing phase. The importance of plaque control and maintenance care cannot be overemphasized, particularly in furcation defects where anatomical complexity may compromise oral hygiene and long-term stability [20]. Despite the favorable findings, certain limitations should be considered. The follow-up period was limited to 6 months, which may not fully reflect the long-term stability of regenerated tissues. Periodontal regeneration should ideally be evaluated over longer periods to assess whether the clinical attachment gain and bone fill remain stable. Another limitation is that only mandibular molar furcation defects were included. Therefore, the findings may not be directly applicable to maxillary furcation defects, which present greater anatomical complexity due to root morphology, furcation entrance dimensions and access limitations [21]. Histological confirmation of true periodontal regeneration was not possible because this was a human clinical study. Therefore, clinical and radiographic improvements were used as surrogate indicators of regeneration. Although these outcomes are valuable and clinically relevant, they cannot definitively confirm the formation of new cementum, periodontal ligament and alveolar bone. Future studies with longer follow-up periods, larger sample sizes, advanced three-dimensional imaging and, where ethically feasible, histological evaluation would provide stronger evidence regarding the quality of regenerated tissues. From a clinical perspective, bioresorbable collagen membrane-assisted GTR appears to be an effective treatment option for Grade II mandibular molar furcation defects. Although the use of a membrane increases treatment cost, the improved clinical outcomes and avoidance of secondary surgery may justify its use in selected patients. Cost-effectiveness should be assessed not only in terms of material expense but also by considering long-term tooth retention, reduced need for retreatment and improved periodontal stability [22]. Overall, the present study supports the use of bioresorbable collagen membranes as a predictable regenerative approach for the treatment of Grade II mandibular molar furcation defects. The test group showed significantly greater probing depth reduction, clinical attachment gain, horizontal probing depth reduction, radiographic bone fill and furcation grade improvement compared with open flap debridement alone. These findings highlight the clinical value of guided tissue regeneration in improving periodontal outcomes when appropriate case selection and meticulous surgical protocols are followed.

## Conclusion:

Guided tissue regeneration using bioresorbable collagen membranes showed significantly superior clinical and radiographic outcomes compared to open flap debridement alone in the management of Grade II mandibular molar furcation defects. The GTR approach resulted in greater probing depth reduction; clinical attachment gain and bone fill over a 6-month period, confirming its effectiveness as a regenerative therapy. These findings support the use of GTR as a preferred treatment modality in appropriately selected cases of Grade II furcation involvement.

## Figures and Tables

**Table 1:** Baseline demographic and clinical characteristics

**Parameter**	**Test Group (n=24)**	**Control Group (n=24)**	**p-value**
Age (years)	45.2 ± 7.9	44.1 ± 8.6	0.648
Gender (M/F)	13/11	13/11	1
Plaque Index	0.82 ± 0.24	0.78 ± 0.21	0.542
Gingival Index	1.14 ± 0.32	1.08 ± 0.28	0.487
Probing Depth (mm)	6.54 ± 0.86	6.38 ± 0.92	0.528
CAL (mm)	7.21 ± 0.94	7.08 ± 0.88	0.621
HPD (mm)	4.42 ± 0.68	4.29 ± 0.72	0.516
Gingival Recession (mm)	0.67 ± 0.48	0.71 ± 0.46	0.762
Values expressed as
mean ± SD;
CAL: Clinical
Attachment Level;
HPD: Horizontal
Probing Depth

**Table 2:** Comparison of clinical parameters at baseline and 6 months

**Parameter**	**Time Point**	**Test Group**	**Control Group**	**p-value\(intergroup)**
Probing Depth (mm)	Baseline	6.54 ± 0.86	6.38 ± 0.92	0.528
	6 months	3.12 ± 0.58	4.21 ± 0.72	<0.001*
	Change	3.42 ± 0.78	2.18 ± 0.65	<0.001*
CAL (mm)	Baseline	7.21 ± 0.94	7.08 ± 0.88	0.621
	6 months	4.32 ± 0.76	5.54 ± 0.82	<0.001*
	Change	2.89 ± 0.72	1.54 ± 0.58	<0.001*
HPD (mm)	Baseline	4.42 ± 0.68	4.29 ± 0.72	0.516
	6 months	1.75 ± 0.52	3.06 ± 0.64	<0.001*
	Change	2.67 ± 0.54	1.23 ± 0.48	<0.001*
Gingival Recession (mm)	Baseline	0.67 ± 0.48	0.71 ± 0.46	0.762
	6 months	1.21 ± 0.42	1.38 ± 0.52	0.218
	Change	0.54 ± 0.38	0.67 ± 0.44	0.274
*Values expressed
as mean ± SD;
statistically
significant (p<0.05)

**Table 3:** Radiographic outcomes and treatment success rates

**Parameter**	**Test Group (n=24)**	**Control Group (n=24)**	**p-value**
Baseline Bone Loss (%)	52.4 ± 14.6	50.8 ± 13.2	0.684
6-month Bone Loss (%)	26.8 ± 11.4	39.4 ± 12.8	<0.001*
Radiographic Bone Fill (%)	48.6 ± 12.3	22.4 ± 9.8	<0.001*
Complete Closure (Grade 0), n (%)	7 (29.2%)	1 (4.2%)	0.018*
Partial Improvement (Grade I), n (%)	14 (58.3%)	10 (41.7%)	0.247
No Improvement, n (%)	3 (12.5%)	13 (54.2%)	0.002*
*Values expressed
as mean ± SD or n (%);
statistically
significant (p<0.05)
